# Mucoadhesive drug delivery systems

**DOI:** 10.4103/0975-7406.76478

**Published:** 2011

**Authors:** Rahamatullah Shaikh, Thakur Raghu Raj Singh, Martin James Garland, A David Woolfson, Ryan F. Donnelly

**Affiliations:** Drug Delivery Group, School of Pharmacy, Queen’s University Belfast, Medical Biology Centre, 97 Lisburn Road, Belfast, BT9 7BL, UK

**Keywords:** Mucoadhesion, mucoadhesive drug delivery systems, mucoadhesive materials

## Abstract

Mucoadhesion is commonly defined as the adhesion between two materials, at least one of which is a mucosal surface. Over the past few decades, mucosal drug delivery has received a great deal of attention. Mucoadhesive dosage forms may be designed to enable prolonged retention at the site of application, providing a controlled rate of drug release for improved therapeutic outcome. Application of dosage forms to mucosal surfaces may be of benefit to drug molecules not amenable to the oral route, such as those that undergo acid degradation or extensive first-pass metabolism. The mucoadhesive ability of a dosage form is dependent upon a variety of factors, including the nature of the mucosal tissue and the physicochemical properties of the polymeric formulation. This review article aims to provide an overview of the various aspects of mucoadhesion, mucoadhesive materials, factors affecting mucoadhesion, evaluating methods, and finally various mucoadhesive drug delivery systems (buccal, nasal, ocular, gastro, vaginal, and rectal).

In the last two decades, mucoadhesion has shown renewed interest for prolonging the residence time of mucoadhesive dosage forms through various mucosal routes in drug delivery applications. Mucoadhesive-based topical and local systems have shown enhanced bioavailability. Mucoadhesive drug delivery gives rapid absorption and good bioavailability due to its considerable surface area and high blood flow. Drug delivery across the mucosa bypasses the first-pass hepatic metabolism and avoiding the degradation of gastrointestinal enzymes. Thus mucosal drug delivery system could be of value in delivering a growing number of high-molecular-weight sensitive molecules such as peptide and oligonucleotides. In this review, the aim is to provide detailed understanding of mucoadhesion, bioadhesion of polymer, and techniques for the determination of mucoadhesion; finally most common routes of mucoadhesive administration will be presented along with examples of formulation studied.

## Bioadhesion and Mucoadhesion

The term bioadhesion can be defined as the state in which two materials, at least one biological in nature, are held together for an extended period of time by interfacial forces.[1] In biological systems, bioadhesion can be classified into 3 types:

- Type 1, adhesion between two biological phases, for example, platelet aggregation and wound healing.
- Type 2, adhesion of a biological phase to an artificial substrate, for example, cell adhesion to culture dishes and biofilm formation on prosthetic devices and inserts.
- Type 3, adhesion of an artificial material to a biological substrate, for example, adhesion of synthetic hydrogels to soft tissues[2] and adhesion of sealants to dental enamel.

For drug delivery purposes, the term bioadhesion implies attachment of a drug carrier system to a specified biological location. The biological surface can be epithelial tissue or the mucus coat on the surface of a tissue. If adhesive attachment is to a mucus coat, the phenomenon is referred to as mucoadhesion. Leung and Robinson[3] described mucoadhesion as the interaction between a mucin surface and a synthetic or natural polymer. Mucoadhesion should not be confused with bioadhesion; in bioadhesion, the polymer is attached to the biological membrane and if the substrate is mucus membrane the term mucoadhesion is used.

## Theories of Mucoadhesion

Various theories exist to explain at least some of the experimental observations made during the bioadhesion process. Unfortunately, each theoretical model can only explain a limited number of the diverse range of interactions that constitute the bioadhesive bond.[4] However, four main theories can be distinguished.

## Wetting Theory of Mucoadhesion

The wetting theory is perhaps the oldest established theory of adhesion. It is best applied to liquid or low-viscosity bioadhesives. It explains adhesion as an embedding process, whereby adhesive agents penetrate into surface irregularities of the substrate and ultimately harden, producing many adhesive anchors. Free movement of the adhesive on the surface of the substrate means that it must overcome any surface tension effects present at the interface.[5] The wetting theory calculates the contact angle and the thermodynamic work of adhesion.

**Figure 1:**
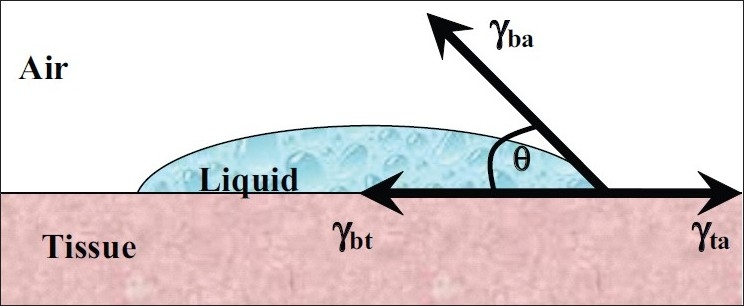
A liquid bioadhesive spreading over a typical soft tissue surface

The work done is related to the surface tension of both the adhesive and the substrate, as given by Dupre’s equation;[6]

(1) $$\omega_{A}=\;\gamma_{b}+\gamma_{t}-\gamma_{b}$$

where ω_A_ is the specific thermodynamic work of adhesion and γ_b_, γ_τ_, and γ_bt_ represent, respectively, the surface tensions of the bioadhesive polymer, the substrate, and the interfacial tension. The adhesive work done is a sum of the surface tensions of the two adherent phases, less the interfacial tensions apparent between both phases.[7] Figure 1 shows a drop of liquid bioadhesive spreading over a soft-tissue surface.

Horizontal resolution of the forces gives the Young equation:

(2) $$\gamma_{ta}=\;\gamma_{bt}+\gamma_{ba}\cos\theta$$

where θ is the angle of contact, γ_bt_ is the surface tension between the tissue and polymer, γ_ba_ is the surface tension between polymer and air, and γ_ta_ is the surface tension between tissue and air. Equation 3 states that if the angle of contact,θ, is greater than zero, the wetting will be incomplete. If the vector γ_ta_ greatly exceeds γ_bt_ + γ_ba_, that is:

(3) $$\gamma_{ta}\geq \;\gamma_{bt}+\gamma_{ba}$$

then θ will approach zero and wetting will be complete. If a bioadhesive material is to successfully adhere to a biological surface, it must first dispel barrier substances and then spontaneously spread across the underlying substrate, either tissue or mucus. The spreading coefficient, *S*_b_, can be defined as shown in Equation 4:

(4) $$S_{b}=\;\gamma_{ta}-\gamma_{bt}-\gamma_{ba\;>0}$$

which states that bioadhesion is successful if *S*_b_ is positive, thereby setting the criteria for the surface tension vectors; in other words, bioadhesion is favored by large values of γ_ta_ or by small values of γ_bt_ and γ_ba_.[7]

## Electrostatic Theory of Mucoadhesion

According to electrostatic theory, transfer of electrons occurs across the adhesive interface and adhering surface. This results in the establishment of the electrical double layer at the interface and a series of attractive forces responsible for maintaining contact between the two layers.[8]

## Diffusion Theory of Mucoadhesion

**Figure 2:**
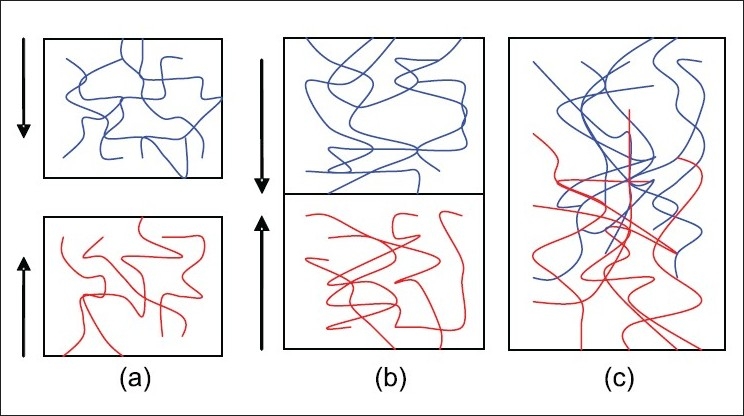
(a) Schematic representation of the diffusion theory of bioadhesion. Blue polymer layer and red mucus layer before contact; (b) Upon contact; (c) The interface becomes diffuse after contact for a period of time

Diffusion theory describes that polymeric chains from the bioadhesive interpenetrate into glycoprotein mucin chains and reach a sufficient depth within the opposite matrix to allow formation of a semipermanent bond.[9] The process can be visualized from the point of initial contact. The existence of concentration gradients will drive the polymer chains of the bioadhesive into the mucus network and the glycoprotein mucin chains into the bioadhesive matrix until an equilibrium penetration depth is achieved as shown in Figure 2.

The exact depth needed for good bioadhesive bonds is unclear, but is estimated to be in the range of 0.2–0.5 *μ*m.[10] The mean diffusional depth of the bioadhesive polymer segments, *s*, may be represented by Equation 5:

(5) $$s=\sqrt{2tD}$$

where *D* is the diffusion coefficient and t is the contact time. Duchene[11] adapted Equation 5 to give Equation 6, which can be used to determine the time, *t*, to bioadhesion of a particular polymer:

(6) $$t=\frac{l^{2}}{D_{b}}$$

in which *l* represents the interpenetrating depth and *D_b_* the diffusion coefficient of a bioadhesive through the substrate.

Once intimate contact is achieved, the substrate and adhesive chains move along their respective concentration gradients into the opposite phases. Depth of diffusion is dependent on the diffusion coefficient of both phases. Reinhart and Peppas[12] reported that the diffusion coefficient depended on the molecular weight of the polymer strand and that it decreased with increasing cross-linking density.

## Adsorption Theory of Mucoadhesion

According to the adsorption theory, after an initial contact between two surfaces, the materials adhere because of surface forces acting between the chemical structures at the two surfaces.[13] When polar molecules or groups are present, they reorientate at the interface.[7] Chemisorption can occur when adhesion is particularly strong. The theory maintains that adherence to tissue is due to the net result of one or more secondary forces (van der Waal’s forces, hydrogen bonding, and hydrophobic bonding).[14–16]

## Fracture Theory of Adhesion

This theory describes the force required for the separation of two surfaces after adhesion. The fracture strength is equivalent adhesive strength through the following equation. This theory is useful for the study of bioadhesion by tensile apparatus.

(7) $$\sigma \;=\;{\left(E\;\times \;\varepsilon /L\right)}^{1/2}$$

where σ is the fracture strength, e fracture energy, E young modulus of elasticity, and L the critical crack length.[17]

## Mucoadhesive Materials

Mucoadhesive polymers have numerous hydrophilic groups, such as hydroxyl, carboxyl, amide, and sulfate. These groups attach to mucus or the cell membrane by various interactions such as hydrogen bonding and hydrophobic or electrostatic interactions. These hydrophilic groups also cause polymers to swell in water and, thus, expose the maximum number of adhesive sites.[16]

An ideal polymer for a bioadhesive drug delivery system should have the following characteristics;[9,13]

1. The polymer and its degradation products should be nontoxic and nonabsorbable.
2. It should be nonirritant.
3. It should preferably form a strong noncovalent bond with the mucus or epithelial cell surface.
4. It should adhere quickly to moist tissue and possess some site specificity.
5. It should allow easy incorporation of the drug and offer no hindrance to its release.
6. The polymer must not decompose on storage or during the shelf life of the dosage form.
7. The cost of the polymer should not be high so that the prepared dosage form remains competitive.

Polymers that adhere to biological surfaces can be divided into three broad categories:[7,10]

1. Polymers that adhere through nonspecific, noncovalent interactions which are primarily electrostatic in nature
2. Polymers possessing hydrophilic functional groups that hydrogen bond with similar groups on biological substrates
3. Polymers that bind to specific receptor sites on the cell or mucus surface

The latter polymer category includes lectins and thiolated polymers. Lectins are generally defined as proteins or glycoprotein complexes of nonimmune origin that are able to bind sugars selectively in a noncovalent manner.[18] Lectins are capable of attaching themselves to carbohydrates on the mucus or epithelial cell surface and have been extensively studied, notably for drug-targeting applications.[19,20] These second-generation bioadhesives not only provide for cellular binding, but also for subsequent endo- and transcytosis. Thiolated polymers, also designated thiomers, are hydrophilic macromolecules exhibiting free thiol groups on the polymeric backbone. Due to these functional groups, various features of polyacrylates and cellulose derivatives were strongly improved.[21] The presence of thiol groups in the polymer allows the formation of stable covalents bonds with cysteine-rich subdomains of mucus glycoproteins leading to increased residence time and improved bioavailability.[22] Other advantageous mucoadhesive properties of thiolated polymers include improved tensile strength, rapid swelling, and water uptake behavior. Table 1 shows the chemical structures of several bioadhesive polymers commonly used in modern drug delivery.

**Table 1:**
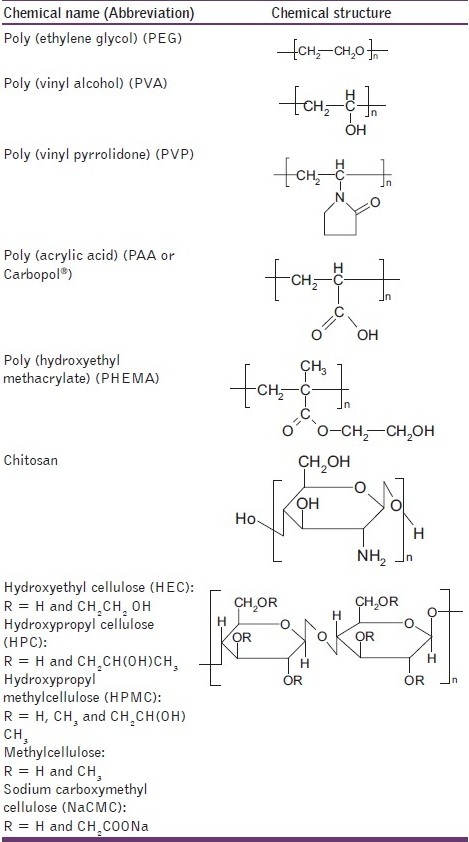
Chemical structures of some bioadhesive polymers used in drug delivery

## Factors Affecting Mucoadhesion

Mucoadhesion may be affected by a number of factors, including hydrophilicity, molecular weight, cross-linking, swelling, pH, and the concentration of the active polymer.[9,13,23]

## Hydrophilicity

Bioadhesive polymers possess numerous hydrophilic functional groups, such as hydroxyl and carboxyl. These groups allow hydrogen bonding with the substrate, swelling in aqueous media, thereby allowing maximal exposure of potential anchor sites. In addition, swollen polymers have the maximum distance between their chains leading to increased chain flexibility and efficient penetration of the substrate.

## Molecular Weight

The interpenetration of polymer molecules is favored by low-molecular-weight polymers, whereas entanglements are favored at higher molecular weights. The optimum molecular weight for the maximum mucoadhesion depends on the type of polymer, with bioadhesive forces increasing with the molecular weight of the polymer up to 100,000. Beyond this level, there is no further gain.[24]

## Cross-linking and Swelling

Cross-link density is inversely proportional to the degree of swelling.[25] The lower the cross-link density, the higher the flexibility and hydration rate; the larger the surface area of the polymer, the better the mucoadhesion. To achieve a high degree of swelling, a lightly cross-linked polymer is favored. However, if too much moisture is present and the degree of swelling is too great, a slippy mucilage results and this can be easily removed from the substrate.[26] The mucoadhesion of cross-linked polymers can be enhanced by the inclusion in the formulation of adhesion promoters, such as free polymer chains and polymers grafted onto the preformed network.[23]

## Spatial Conformation

Besides molecular weight or chain length, spatial conformation of a polymer is also important. Despite a high molecular weight of 19,500,000 for dextrans, they have adhesive strength similar to that of polyethylene glycol (PEG), with a molecular weight of 200,000. The helical conformation of dextran may shield many adhesively active groups, primarily responsible for adhesion, unlike PEG polymers, which have a linear conformation.[9]

## pH

The pH at the bioadhesive to substrate interface can influence the adhesion of bioadhesives possessing ionizable groups. Many bioadhesives used in drug delivery are polyanions possessing carboxylic acid functionalities. If the local pH is above the p*K* of the polymer, it will be largely ionized; if the pH is below the p*K* of the polymer, it will be largely unionized. The approximate p*K*^a^ for the poly(acrylic acid) family of polymers is between 4 and 5. The maximum adhesive strength of these polymers is observed around pH 4–5 and decreases gradually above a pH of 6. A systematic investigation of the mechanisms of mucoadhesion clearly showed that the protonated carboxyl groups, rather than the ionized carboxyl groups, react with mucin molecules, presumably by the simultaneous formation of numerous hydrogen bonds.[27]

## Concentration of Active Polymer

Ahuja[10] stated that there is an optimum concentration of polymer corresponding to the best mucoadhesion. In highly concentrated systems, beyond the optimum concentration the adhesive strength drops significantly. In concentrated solutions, the coiled molecules become solvent-poor and the chains available for interpenetration are not numerous. This result seems to be of interest only for more or less liquid mucoadhesive formulations. It was shown by Duchêne[11] that, for solid dosage forms such as tablets, the higher the polymer concentration, the stronger the mucoadhesion.

## Drug/Excipient Concentration

Drug/excipient concentration may influence the mucoadhesion. BlancoFuente[28] studied the effect of propranolol hydrochloride to Carbopol^®^ (a lightly cross-linked poly(acrylic acid) polymer) hydrogels adhesion. Author demonstrated increased adhesion when water was limited in the system due to an increase in the elasticity, caused by the complex formation between drug and the polymer. While in the presence of large quantities of water, the complex precipitated out, leading to a slight decrease in the adhesive character. Increasing toluidine blue O (TBO) concentration in mucoadhesive patches based on Gantrez^®^ (poly(methylvinylether/maleic acid) significantly increased mucoadhesion to porcine cheek tissue.[29] This was attributed to increased internal cohesion within the patches due to electrostatic interactions between the cationic drug and anionic copolymer.

## Other Factors Affecting Mucoadhesion

Mucoadhesion may be affected by the initial force of application.[30] Higher forces lead to enhanced interpenetration and high bioadhesive strength.[11] In addition, the greater the initial contact time between bioadhesive and substrate, the greater the swelling and interpenetration of polymer chains.[31] Physiological variables can also affect mucoadhesion. The rate of mucus turnover can be affected by disease states and also by the presence of a bioadhesive device.[32] In addition, the nature of the surface presented to the bioadhesive formulation can vary significantly depending on the body site and the presence of local or systemic disease.[31]

## Techniques for the Determination of Mucoadhesion

The evaluation of bioadhesive properties is fundamental to the development of novel bioadhesive delivery systems. These tests are also important to screen large number of materials and their mechanisms. Numerous methods have been developed for studying mucoadhesion. Since no standard apparatus is available for testing bioadhesive strength, an inevitable lack of uniformity between test methods has arisen. Nevertheless, three main testing modes are recognized – tensile test, shear strength, and peel strength.

The most popular technique used for the determination of force of separation in bioadhesive testing is the application of force perpendicularly to the tissue/adhesive interface, during which a state of tensile stress is set up. But during the shear stress, the direction of the forces is reoriented so that it acts along the joint interface. In both tensile and shear modes, an equal pressure is distributed over the contact area.[33]

The peel test is based on the calculation of energy required to detach the patch from the substrate. The peel test is of limited use in most bioadhesive systems. However, it is of value when the bioadhesive system is formulated as a patch.[34]

In tensile and shear experiments, the stress is uniformly distributed over the adhesive joint, whereas in the peel strength stress is focused at the edge of the joint. Thus tensile and shear measure the mechanical properties of the system, whereas peel strength measures the resistant of the peeling force.

**Figure 3:**
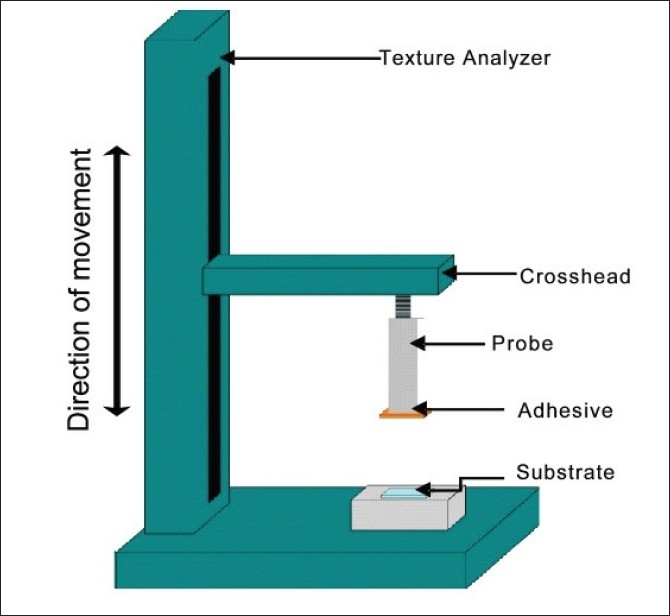
Texture profile analyzer in bioadhesion test mode

Review of the literature confirmed that the most common technique used for the measurement of bioadhesion test is tensile strength method. McCarron *et al*.[26,34,35] and Donnelly[36] have reported extensively on the use of a commercial apparatus, in the form of a texture profile analyzer [Figure 3] operating in bioadhesive test mode, to measure the force required to remove bioadhesive films from excised tissue *in vitro*.

**Figure 4:**
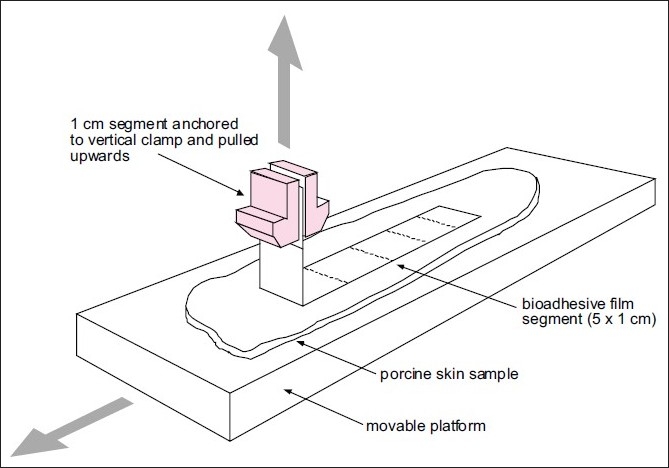
Simplified representation of a typical test set-up used to determine peel strength of bioadhesive films

The texture analyzer, operating in tensile test mode and coupled with a sliding lower platform, was also used to determine peel strength of similar formulations [Figure 4].[34]

Rheological techniques that study the flow and deformation of materials may be useful in predicting the mucoadhesive ability of a polymeric formulation. A simple rheological approach for polymer solutions and gels was first suggested by Hassan and Gallo.[37] In this method, rheological interaction between a polymer gel and mucin solution was determined. It was shown that a polymer gel and mucin solution mixture exhibited larger rheological responsethan the sum of the values of polymer and mucin. However, a wide variation in results is found in the literature that utilize rheological methods for mucoadhesion determination, which may be attributable to differences in mucin type and concentration,[38,39] as well as polymer concentrations.[40,39] Therefore, Hagerstrom[41] recommend that the rheological method should not be used as a stand-alone method for studying the mucoadhesive properties of the polymer gels.

*In vivo* aspects of mucoadhesive testing have recently been reported to monitor the mucoadhesion on tissue surface such as the GIT or the buccal cavity. However, there are only a limited number of *in vivo* studies reported in the literature *in vitro* work because of the time, cost, and ethical constrains. The most common *in vivo* techniques to monitor mucoadhesion include GI transit times of bioadhesive-coated particles and drug release from *in situ* bioadhesive devices.

Ch’ng[42] studied the *in vivo* transit time for bioadhesive beads in the rat GIT. A 51Cr-labeled bioadhesive was inserted at selected time intervals; the GITs were removed. The GIT of the rat was then cut into 20 equal segments and the radioactivity was measured.

Davis[43] investigated the noninvasive *in vivo* technique to determine the transit of mucoadhesive agent. Therefore, in this study a formulation was used containing a gamma-emitting radionuclide. The release characteristics and the position polymer could be examined by gamma scintigraphy.

In recent times, magnetic resonance imaging (MRI) is another noninvasive technique that is widely used. Christian Kremser[44] used MRI to detect the time and location of release of mucoadhesive formulation using dry Gd-DOTA powder.

## Routes of Administration for Mucoadhesive-based Drug Delivery Systems

Mucosa or the mucus membrane is the moist tissue that lines organs and body cavities such as mouth, gut, rectum, genital area, nose, and eye lid. Anatomical differences of the mucus membrane at varying body locations are given in Table 2. Mucoadhesive drug delivery systems in the past have been formulated as powders, compacts, sprays, semisolids, or films. For example, compacts have been used for drug delivery to the oral cavity,[51] and powders and nanoparticles have been used to facilitate drug administration to the nasal mucosa.[52,53] Recently oral strips[54] were developed for tongue or buccal cavity. Details of the mucoadhesive dosage forms are given in Table 3. Recently, there has been a growing interest in alternative delivery system designs. Buccal films have been suggested as a means of offering greater flexibility and comfort than adhesive tablets. In addition, films may circumvent the problem of the relatively short residence time of oral gels.[77] Film-forming bioadhesive polymers used in the production of bioadhesive films include the cellulose derivatives,[77] poly(acrylic acids) such as Carbopol,^®^^78^] and Gantrez^®^ copolymers such as poly(methylvinylether/maleic anhydride).[45]

**Table 2:** Anatomical differences of the mucus membrane

Mucus membrane	Relevant anatomical features
Buccal[45]	Buccal mucosa surface area approximately 30 cm^2^
	Comprised of three distinct layers – epithelium, basement membrane, and connective tissues
	Buccal mucosa, sublingual are soft palate nonkeratinized tissue, and gingival are hard palate keratinized tissue
	Thickness of buccal epithelium is in the range of 500–800 *μ*m, 40–50 cells thick
	Mucus secreted by salivary glands, as a component of saliva, forming a 0.1–0.7 mm thick layer
	Turnover time for buccal epithelium 5–6 days
	Permeability barrier property of oral mucosa due to intercellular materials derived from membrane-coating granules
Nasal[46]	Nasal cavity surface area 160 cm^2^
	Lined with mucous membrane containing columnar cells, goblet cells, and basal cells
	Columnar cells are covered with cilia, apart from the anterior part of the nasal cavity
	Both keratinized and nonkeratinized epithelial cells present depending upon location within nasal cavity
	Cilia responsible for mucociliary clearance
	Mucus secreted by the submucosal glands and the goblet cells, forming a mucus layer approximately 5–20 *μ*m thick
	Nasal cavity length approximately 60 mm
	Nasal cavity volume approximately 20 mL
	Turn-over time for mucus is usually 10–15 min
Ocular[47]	Cornea is composed of five layers – epithelium, Bowman’s layer, stroma, Descemet’s membrane, and endothelium
	Epithelium consists of 5–6 layers of cells, with the cells of the basal layer being columnar, and the outermost cells flattened polygonal cells
	Tight junctions present between the basal cells of the corneal epithelium
	At the corneal margin, the conjunctiva is structurally continuous with the corneal epithelium
	The conjunctival tissue is permeable to molecules up to 20,000 Da, whereas the cornea is impermeable to molecules greater than 5000 Da
	The conjunctiva contains around 1.5 million goblet cells, which synthesize secretory mucins and peptides
	A volume of about 2–3 *μ*L of mucus os secreted daily
	A turnover of the mucus layer occurs in approximately 15–20 h
	Exposed part of the eye is covered by a thin fluid layer – percorneal tear film
Mucus Membrane	Relevant Anatomical Features
	Tear film thickness is approximately 3–10 *μ*m
Vaginal[48]	Length of vagina varies from 6 to 10 cm
	The epithelial layer consists of the lamina propia and stratified squamous epithelium
	A cell turnover of about 10–15 layers is estimated to be in the order of 7 days
	Although there are no glands in the vagina mucosa, the surface is usually covered with vaginal fluid
	Major components of vaginal fluid are cervical mucus and vaginal fluid from the well-vascularized mucosa
	The volume, viscosity, and pH of the cervical mucus vary with age and during the menstrual cycle
Rectal[49]	Length approximately 15–20 cm
	Surface area of approximately 300 cm^2^
	Epithelium consists of a single layer of cylindrical cells and goblet cells secreting mucus
	Flat surface, without villi, and with three major fold, the rectal valves
	Approximately 3 mL of mucus with a neutral pH spread over the surface

**Table 3:** Different types of mucoadhesive dosage forms

Delivery routes	Dosage forms				
	Tablet	Ointment	Gel	Patch	Film
Buccal	Theophylline, multiple polymers[55]	Benzyl nicotinate, multiple polymers[56]	Benzydamine, chitosan derivatives[57]	Miconazole, PVA/PVP[58]	Fentanyl, PVP[59]
Nasal	N/A	Mupirocin, glycerin ester[60]	Insulin, starch[61]	Insulin, chitosan/PEG[62]	Chlorpromazine, chitosan/pectin[63]
Ocular	Diclofenac, poly(acrylic) acid[64]	Sulphadicramide, multiple polymers[65]	Puerarin, poloxamer/carbopol[66]	Ciprofloxacin, PVA/CMC[67]	Fluorescein, HPMC[68]
Vaginal	Metronidazole, chitosan[69]	Terameprocol, white petroleum[70]	Amphotericin, pluronic[71]	ALA, PMVE/MA[34]	SDS, multiple polymers[72]
Rectal	Ramosetron, carbopol[73]	Zinc oxide, petroleum[74]	Quinine, HPMC[75]	N/A	Theophylline, pHEMA[76]

## Oral Mucoadhesive Drug Delivery Systems

Drug delivery through the oral mucosa has gained significant attention due to its convenient accessibility. The buccal and sublingual routes are considered as the most commonly used rotes. The nonkeratinized epithelium in the oral cavity, such as the soft palate, the mouth floor, the ventral side of the tongue, and the buccal mucosa, offers a relatively permeable barrier for drug transport.[79] Hydrophilic compounds and large or highly polar molecules follow paracellular transport, whereas transcellular transport through the lipid bilayer is followed by lipophilic drugs.[80] Drug delivery through the oral mucosa has proven particularly useful and offers several advantages over other drug delivery systems including bypassing hepatic first-pass metabolism, increasing the bioavailability of drugs, improved patient compliance, excellent accessibility, unidirectional drug flux, and improved barrier permeability compared, for example, to intact skin.[81,82] The oral cavity has been used as a site for local and systemic drug delivery. Local drug therapy is used to treat disease states like aphthous ulceration gingivitis, periodontal disease, and xerostoma. Different dosage designs include adhesive gels, tablets, films, patches, ointments, mouth washes, and pastes.

Until now adhesive tablets have been the most commonly used dosage forms for buccal drug delivery. Tablets can be applied to different regions of oral cavity, such as cheeks, lips, gums, and palate. Unlike conventional tablets, buccal tablets allow drinking, eating, and speaking without any major discomfort. Perioli[83] studied the influence of compression force on tablet behavior and drug release rate for mucoadhesive buccal tablets. Tablets were prepared by using hydroxyethyl cellulose (HEC) and carbopol 940 in a 1:1 ratio as matrix-forming polymers at varying compression forces. Compression forces did not significantly affect the water penetration and polymer chain stretching; however, mucoadhesion performance and drug release were influenced by compression force. Increase in compression force resulted in a decreased *in vitro* and *in vivo* drug release while giving the best *in vivo* mucoadhesive and hydration time. Moreover, it was observed that tablets prepared with the lowest force gave the best results, in comparison with tablets prepared with highest forces causing pain during *in vivo* application, needing to be detached by human volunteers.

Oral mucosal ulceration is a common condition with up to 50% of healthy adults suffering from recurrent minor mouth ulcers (aphthous stomatitis). Shermer[84] evaluated the efficacy and tolerability of a mucoadhesive patch compared with a pain-relieving oral solution for the treatment of aphthous stomatitis. The mucoadhesive patch was found to be more effective than the oral solution in terms of healing time and pain intensity after 12 and 24 h. Local adverse effects 1 h after the treatment were significantly less frequent among the mucoadhesive patch patients compared with the oral solution patients.

Donnelly[29] reported on a mucoadhesive patch containing TBO as a potential delivery system for use in photodynamic antimicrobial chemotherapy (PACT) of oropharyngeal candidiasis. Patches are prepared from aqueous blends of poly(methyl vinyl ether/maleic anhydride) and tripropyleneglycol methyl ether. The authors concluded that short application times of TBO-containing mucoadhesive patches should allow the treatment of recently acquired oropharyngeal candidiasis, caused solely by planktonic cells. Longer patch application times may be required for persistent disease where biofilms are implicated.

Periodontitis is an inflammatory disease of the oral cavity, which results in the destruction of the supporting structures of the teeth.[85] Inflammatory periodontitis disease can be treated by the combination of mechanical and intraperiondontal pocket chemotherapeutic agents.[86] Jones and Andrews[87,88] described the formulation and physicochemical characterization of syringeable semisolid, bioadhesive networks (containing tetracycline, metronidazole, or model protein drugs). Such systems may be formulated to exhibit requisitory flow properties (and hence may be easily administered into the periodontal pocket using a syringe), mucoadhesive properties (ensuring prolonged retention within the pocket), and sustained release of therapeutic agent within this environment.

Mucosal delivery of drugs *via* the buccal route is still very challenging in spite of extensive clinical studies. Here, we are underlining several formulations which are in clinical trials or commercial products. The 3M company has developed a buccal patch system which consists of a matrix patch containing drug, mucoadhesive polymers, and polymeric elastomers surrounded by a backing material. Their buprenorphine patch is capable of delivering the drug for a period up to 12 h, with good patient comfort reported.[89]

Oralin, a novel liquid aerosol formulation (Generex Biotechnology), has been developed and it is now in clinical phase II trials.[90] Oralin allows precise insulin dose delivery *via* a metered dose inhaler in the form of fine aerosolized droplets directed into the mouth. Levels of drug in the mouth are noticeably increased compared with conventional formulations. This oral aerosol formulation is rapidly absorbed through the buccal mucosal epithelium, and it provides the plasma insulin levels necessary to control postprandial glucose rise in diabetic patients. This novel, pain-free, oral insulin formulation has a number of advantages, including rapid absorption, user-friendly administration technique, precise dosing control (comparable to injection within one unit), and bolus delivery of drug. Furthermore, BioAlliance Pharma’s miconazole tablet (Lauriad^®^) formulation is now in clinical phase III trials, and Aphtach^®^ (triamcinolone acetonide buccal tablets from Teijin Ltd.) are now commercially available.[90]

## Nasal Mucoadhesive Drug Delivery Systems

The area of the normal human nasal mucosa is approximately 150 cm^2^, a highly dense vascular network and relatively permeable membrane structure; all these factors make nasal cavity interesting.[91] Drawbacks are local toxicity/irritation mucociliary clearance of 5 min, presence of proteolytic enzymes, and influence of pathological conditions (cold and allergies). Among the advantages are rapid uptake and avoiding first-pass hepatic metabolism. In addition, bioadhesive application of liquids, semisolids, and solids can significantly increase retention time.

Nasal delivery of protein and peptide therapeutics can be compromised by the brief residence time at this mucosal surface. Some bioadhesive polymers have been suggested to extend residence time and improve protein uptake across the nasal mucosa. McInnes[92] quantified nasal residence of bioadhesive formulations using gamma scintigraphy and investigated absorption of insulin. A four-way crossover study was conducted in six healthy male volunteers, comparing a conventional nasal spray solution with three lyophilized nasal insert formulations (1–3% w/w hydroxypropylmethyl cellulose, HPMC). The authors concluded that the 2% w/w HPMC lyophilized insert formulation achieved extended nasal residence, demonstrating an optimum combination of rapid adhesion without overhydration.

Coucke[93] studied viscosity-enhancing mucosal delivery systems for the induction of an adaptive immune response against viral antigen. Powder formulations based on spray-dried mixtures of starch (Amioca^®^) and poly(acrylic acid) (Carbopol^®^ 974P) in different ratios were used as carriers of the viral antigen. A comparison of these formulations for intranasal delivery of heat-inactivated influenza virus combined with LTR 192G adjuvant was made *in vivo* in a rabbit model. The authors concluded that the use of bioadhesive carriers based on starch and poly(acrylic acid) facilitates the induction of a systemic anti-HA antibody response after intranasal vaccination with a whole virus influenza vaccine.

Functionalized mucoadhesive polymers, such as polycarbophil, hyaluronan, and amberlite resin, have been developed and the characterization and safety aspects of nasal drug products extensively studied. Recently, mucosal vaccines have been introduced in immunization to induce a systemic immune response. Addition of mucoadhesive polymer to the vaccine formulation increases the affinity for mucus membranes and may enhance the stability of the preparation. Examples of these include intranasal vaccines against influenza, diphtheria, and tetanus.[94]

Pilot studies involving the use of a nasal morphine–chitosan formulation for the treatment of breakthrough pain in 14 cancer patients suggested that this system was acceptable, well-tolerated, and may lead to rapid onset of pain relief.[95]

Tzachev[96] has compared a mucoadhesive solution (formulation of xylometazoline) with commercially available decongestatnt solution in 20 human subjects with perennial allergic rhinitis. The author concluded that the mucoadhesive formulation exhibited a significantly more prolonged clinical effect than the nonmucoadhesive product.

## Ocular Mucoadhesive Drug Delivery Systems

Drug administration to the eye is a challenge because there are several mechanisms (tear production, tear flow, and blinking) that protect the eye from the harmful agents. Conventional delivery methods are not ideal. Solutions and suspensions are readily washed from the cornea and ointments alter the tear refractive index and blur vision; so it is a target to prolong the residence time by mucoadhesion.

Sensoy[97] aimed to prepare bioadhesive sulfacetamide sodium microspheres to increase residence time on the ocular surface and to enhance treatment efficacy of ocular keratitis. Microspheres were fabricated by a spray-drying method using a mixture of polymers, such as pectin, polycarbophil, and HPMC at different ratios. Author concluded that a sulfacetamide sodium–loaded polycarbophil microsphere formulation with a polymer:drug ratio of 2:1 was found to be the most suitable for ocular application and used in *in vivo* studies on New Zealand male rabbit eyes with keratitis caused by *Pseudomonas aeruginosa* and *Staphylococcus aureus*.

Gene transfer is considered to be a promising alternative for the treatment of several chronic diseases that affect the ocular surface. De la Fuente[98] investigated the efficacy and mechanism of action of a bioadhesive DNA nanocarrier made of hyaluronan (HA) and chitosan (CS), specifically designed for topical ophthalmic gene therapy. The author concluded that on topical administration to rabbits, the nanoparticles entered the corneal and conjunctival epithelial cells and got assimilated by the cells. More importantly, the nanoparticles provided an efficient delivery of the associated plasmid DNA inside the cells, reaching significant transfection levels.

Many clinical studies have been performed on mucoadhesive ocular dosage forms. Ocular films applied behind the eye lid were found to prolong retention time and precision of dosing. However, films were found to have a tendency to move across the surface of the eye, thus resulting in irritation, for example, from Ocusert^®^ (Alza). It has been shown that the addition of mucoadhesive polymers to ocular films reduced film movement across the eye, minimizing ocular irritation and burning sensations.[94]

Baeyens[99] conducted a clinical study in dogs presenting with external ophthalmic diseases (conjunctivitis, superficial corneal ulcer, or keratoconjuctivitissicca) using soluble bioadhesive ophthalmic drug inserts (BODI^®^) in comparison with classical Tiacil^®^ eye drops from Virbac Laboratories. The results of the clinical study showed that BODI^®^ demonstrated an advantage over the Tiacil^®^ by reducing the treatment to a single application and, therefore, improving patient compliance.

Mucoadhesive polymers have been incorporated into ophthalmic gels to increase gel efficacy, such as NyoGel ^®^ (timolol, Novartis) and Pilogel^®^ (pilocarpine hydrochloride, Alcon Laborataries).[100]

## Vaginal Mucoadhesive Drug Delivery Systems

The vagina is a fibrovascular tube connecting the uterus to the outer surface of the body. The vaginal epithelium consists of a stratified squamous epithelium and lamina propia. Dosage forms used for vaginal route are solutions, gels, suspensions, suppositories, creams, and tablets and all have short residence time.[101,102,103] Bioadhesives can control the rate of drug release from, and extend the residence time of, vaginal formulations. These formulations may contain drug or, quite simply, act in conjunction with moisturizing agents as a control for vaginal dryness.

Alam[104] developed an acid-buffering bioadhesive vaginal clotrimazole (antifungal) and metronidazole (antiprotozoal and antibacterial) tablets for the treatment of genitourinary tract infections. From bioadhesion experiment and release studies, it was found that polycarbophil and sodium carboxymethyl cellulose was a good combination for an acid-buffering bioadhesive vaginal tablet. From *ex vivo* retention studies, it was found that the bioadhesive polymers held the tablet for more than 24 h inside the vagina. The cumulative release profile of the developed tablet was matched with a marketed conventional tablet (Infa-V^®^). The *in vitro* spreadability of the swelled tablet was comparable to the marketed gel. In the *in vitro* antimicrobial study, it was found that the acid-buffering bioadhesive tablet produced better antimicrobial action than marketed intravaginal drug delivery systems (Infa-V^®^, Candid-V^®^, and Canesten^®^ 1).

Cevher[105] aimed to prepare clomiphene citrate (CLM) gel formulations for the local treatment of human papilloma virus infections. In this respect, 1% w/w CLM gels including polyacrylic acid (PAA) polymers such as Carbopol^®^ 934P (C934P), Carbopol^®^ 971P (C971P), Carbopol^®^ 974P (C974P) in various concentrations, and their conjugates containing thiol groups, were prepared. Author concluded that gels containing C934P-Cys showed the highest adhesiveness and mucoadhesion. A significant decrease was observed in drug release from gel formulations as the polymer concentration increased.

Recent advances in polymeric technology have increased the potential of vaginal gels. Vaginal gels are semisolid polymeric matrices comprising small amounts of solid, dispersed in relatively large amounts of liquid and have been used in systems for microbicides, contraceptives, labor inducers, and other substances.

Several clinical trials are in underway on microbicidal gels. Microbicidal gels are intended to improve mucosal permeation rate of microbicides for the prevention of sexually transmitted diseases. A 1% tenofovir gel is being investigated in phase II clinical trials for determining the safety and acceptability of vaginal microbicides.[106]

Various clinical trials of contraceptive gels are also ongoing, with a view to determine their effectiveness. BufferGel^®^ is in phases II and III clinical trial comparing itto the Gynol II ^®^ marketed product.[106]

Pharmacia conducted clinical trials of the Prostin E2^®^ suppository containing dinoprostone, and found that administration of prostaglandin E2 gel showed to be more effective in inducing labor.[106]

Janssen Pharmaceutica conducted phase III clinical trial of mucoadhesive systems based on itraconazole vaginal cream containing cyclodextrins and other ingredients. Clinical investigation indicated that application of 5 g of 2% cream was well tolerated and was found to be an effective delivery system for selective vaginal delivery.[107]

## Rectal Mucoadhesive Drug Delivery Systems

The rectum is part of the colon, it is 10 cm in length, and has surface area 300 cm^2^. The function of the rectum is mostly concerned with removing water. Surface area without villi gives it a relatively small surface area for drug absorption.[54] Most rectal absorption of drugs is achieved by a simple diffusion process through the lipid membrane. Drugs that are liable to extensive first-pass metabolism can benefit greatly if delivered to the rectal area, especially if they are targeted to areas close to the anus. Furthermore, addition of bioadhesive polymer the migration distance in the rectum decreased.

Kim[108] aimed to develop a thermoreversible flurbiprofen liquid suppository base composed of poloxamer and sodium alginate for the improvement of rectal bioavailability of flurbiprofen. Cyclodextrin derivatives, such as alpha-, beta-, gamma-cyclodextrin, and hydroxypropyl-beta-cyclodextrin (HP-beta-CD), were used to enhance the aqueous solubility of flurbiprofen. Pharmacokinetic studies were performed after rectal administration of flurbiprofen liquid suppositories with and without HP-beta-CD or after intravenous administration of a commercially available product (Lipfen^®^, flurbiprofen axetil-loaded emulsion) to rats. Flurbiprofen liquid suppository containing HP-beta-CD showed an excellent bioavailability in that the AUC of flurbiprofen after its rectal administration was not significantly different from that after intravenous administration of Lipfen^®^. The authors concluded that HP-beta-CD could be a preferable solubility enhancer for the development of liquid suppositories containing poorly water-soluble drugs.

## Cervical and Vulval Drug Delivery Systems

A novel bioadhesive cervical patch containing 5-fluorouracil for the treatment of cervical intraepithelial neoplasia (CIN) was described by Woolfson.[109] This patch was a bilaminar design, with a drug-loaded bioadhesive film cast from a gel containing 2% w/w Carbopol^®^ 981 plasticized with 1%w/w glycerine; the casting solvent was ethanol:water 30:70. The film, which was mechanically stable on storage under ambient conditions, was bonded directly to a backing layer formed from thermally cured poly(vinyl chloride) emulsion. Release of 5-fluorouracil from the bioadhesive layer into an aqueous sink was rapid but was controlled down to an undetectable level through the backing layer. Despite the relatively hydrophilic nature of 5-fluorouracil, substantial drug release through human cervical tissue samples was observed over approximately 20 h.[110]

Donnelly[111] described the design, physicochemical characterization, and clinical evaluation of bioadhesive drug delivery systems for photodynamic therapy of difficult-to-manage vulval neoplasias and dysplasias. Aminolevulic acid (ALA) is commonly delivered to the vulva using creams or solutions, which are covered with an occlusive dressing. Such dressings are poor at staying in place at the vulva, where shear forces are high in mobile patients. To overcome the problems, the authors produced a bioadhesive patch by a novel laminating procedure. The ALA loading was 38 mg cm ^−2^ . Patches were shown to release more ALA over 6 h than the proprietary cream (Porphin^®^, 20% w/w ALA). Clinically, the patch was extensively used in successful PDT of vulval intraepithelial neoplasia, lichen sclerosus, squamous hyperplasia, Paget’s disease, and vulvodynia.

## Gastrointestinal Mucoadhesive Drug Delivery Systems

Oral route is undoubtedly most favored route of administration, but hepatic first-pass metabolism, degradation of drug during absorption, mucus covering GI epithilia, and high turnover of mucus are serious concerns of oral route. In recent years, the gastrointestinal tract (GIT) delivery emerged as a most important route of administration. Bioadhesive retentive system involves the use of bioadhesive polymers, which can adhere to the epithelial surface in the GIT. Using bioadhesive would be achieved increase GI transit time and increase in bioavailability.

Ahmed[112] studied gastric retention formulations (GRFs) made of naturally occurring carbohydrate polymers and containing riboflavin *in vitro* for swelling and dissolution characteristics as well as in fasting dogs for gastric retention. The bioavailability of riboflavin, from the GRFs was studied in fasted healthy humans and compared to an immediate release formulation. It was found that when the GRFs were dried and immersed in gastric juice, they swelled rapidly and released their drug payload in a zero-order fashion for a period of 24 h. *In vivo* studies in dogs showed that a rectangular shaped GRF stayed in the stomach of fasted dogs for more than 9 h, then disintegrated and reached the colon in 24 h. Considering pharmacokinetic parameters of human subjects under fasting conditions, bioavailability of riboflavin from a large size GRF was more than triple of that measured after administration of an immediate release formulation.

Salman[113] aimed to develop polymeric nanoparticulate carriers with bioadhesive properties and to evaluate their adjuvant potential for oral vaccination. Thiamine was used as a specific ligand–nanoparticle conjugate (TNP) to target specific sites within the gastrointestinal tract, namely enterocytes and Peyer’s patches. The affinity of nanoparticles to the gut mucosa was studied in orally inoculated rats. The authors concluded that thiamine-coated nanoparticles showed promise as particulate vectors for oral vaccination and immunotherapy.

## Conclusion

The mucoadhesive dosage forms offer prolonged contact at the site of administration, low enzymatic activity, and patient compliance. The formulation of mucoadhesive drug delivery system depends on the selection of suitable polymer with excellent mucosal adhesive properties and biocompatibility. Now researchers are looking beyond traditional polymers, in particular next-generation mucoadhesive polymers (lectins, thiols, etc.); these polymers offer greater attachment and retention of dosage forms. However, these novel mucoadhesive formulations require much more work, to deliver clinically for the treatment of both topical and systemic diseases.
